# Traditional Chinese medicine acupoint pasting for preventing and treating gastrointestinal reactions in type II diabetes mellitus patients undergoing glucagon-like peptide-1 receptor agonist therapy: A clinical study

**DOI:** 10.5937/jomb0-55092

**Published:** 2025-09-05

**Authors:** Songsong Zheng, Diyi Zhou, Fangfang Chen, Jiandi Zheng

**Affiliations:** 1 Zhejiang Hospital of Integrated Traditional Chinese and Western Medicine, Department of Endocrine and Metabolic Disease, Hangzhou Red Cross Hospital, Hangzhou, Zhejiang Province, China

**Keywords:** T2DM, GLP-1 receptor agonists, gastrointestinal adverse reactions, glycemic control, insulin, T2DM, agonisti GLP-1 receptora, gastrointestinalne neželjene reakcije, kontrola glikemije, insulin

## Abstract

**Background:**

Patients with type 2 diabetes mellitus (T2DM) initiating treatment with glucagon-like peptide-1 receptor agonists (GLP-1RAs) may encounter various risks and complications.

**Methods:**

This study randomly assigned 315 patients starting GLP-1RA therapy into a control group (CG, standard treatment + routine care), a placebo group (PG, placebo + routine care), and a traditional Chinese medicine (TCM) group (TG, TCM plaster therapy + routine care). The glycemic control, pancreatic function, hematological parameters, renal function, and adverse reactions (ARs) were compared among the groups.

**Results:**

The TG exhibited no significant differences in fasting blood glucose (FBG), postprandial blood glucose at two hours, glycated hemoglobin (Hb), fasting insulin (FINS) levels, b-cell insulin secretion, and insulin resistance (IR) when compared to the CG and the PG (P>0.05). Additionally, there were no significant changes in Hb, white blood cell (WBC) count, and erythrocyte sedimentation rate (ESR) in the TG relative to the CG and PG (P>0.05). Renal function indicators revealed that the levels of blood urea nitrogen (BUN) and serum creatinine (Cr) in the TG did not differ significantly from those in the CG and the PG (P>0.05). The incidence of ARs in TG (8.57%) was markedly lower than in PG (17.14%) and CG (18.1%) (P<0.05).

**Conclusions:**

The application of TCM Liangfu Pills via acupoint plaster did not demonstrate significant therapeutic effects on glycemic control, pancreatic function, or routine blood parameters. However, it was effective in significantly reducing the risk of gastrointestinal adverse reactions associated with GLP-1RA therapy.

## Abstract (sr)

**Uvod:**

Ova studija je procenjivala nivoe glikoziliranog hemoglobina (HbA1c), insulina natašte (FINS) i sekrecije insulina b-ćelija (HOMA-b) u serumu kod pacijenata sa dijabetesom tipa II koji su na terapiji agonistima receptora glukagonu sličnog peptida-1 (GLP-1RA).

**Metode:**

Ukupno 315 pacijenata koji su započeli GLP-1RA terapiju je nasumično raspoređeno u tri grupe: kontrolnu grupu (CG), koja je dobijala standardni tretman i rutinsku negu; placebo grupu (PG), koja je pored rutinske nege dobijala placebo; i grupu tradicionalne kineske medicine (TCM) (TG), koja je pored rutinske nege primala i TCM flaster terapiju. Procenjivani su i upoređivani kontrola glikemije, funkcija pankreasa, hematološki parametri, bubrežna funkcija i neželjene reakcije (NR).

## Introduction

With the development of the socio-economic landscape and changes in lifestyle, the incidence of type 2 diabetes mellitus (T2DM) has been rising annually, posing a significant public health challenge globally [1] [2]. According to statistics from the International Diabetes Federation, the number of individuals with T2DM worldwide has exceeded 400 million, and this figure is projected to reach 700 million by 2045. Patients often experience a variety of symptoms, such as extreme thirst, frequent urination, fatigue, and blurred vision, which severely impact their daily lives and work [3]. Additionally, T2DM presents considerable treatment challenges. Due to progressive insulin (INS) resistance and declining secretion function, continuous monitoring of blood glucose (BG) levels is required throughout the treatment process, necessitating adjustments to medication regimens based on individual circumstances [4]. Even with the use of multiple medications, many patients still struggle to achieve optimal glycemic control. Prolonged hyperglycemia can lead to various complications, including cardiovascular diseases, kidney disease, retinopathy, and neuropathy, which not only diminish patients’ quality of life but also increase the burden on healthcare systems [5] [6]. Therefore, it is crucial to seek effective treatment options and management strategies.

Withdrawal and Termination Criteria: (1) Patients who requested to withdraw for any reason; (2) Patients who violated the clinical observation protocol, with missing primary assessments or incomplete data that affect the evaluation of efficacy or safety; (3) Patients who experienced serious ARs related to the study; (4) Patients who began using medications that regulate gastrointestinal function during the study.

## Materials and methods

### Study subjects

A total of 315 patients who were hospitalized in the Department of Endocrinology and Metabolic Diseases at our hospital from July 2021 to October 2023 were selected. These patients met the 2020 edition of the Chinese Diabetes Prevention and Treatment Guidelines for the diagnosis of type 2 diabetes and were using GLP-1 receptor agonists (GLP-1RA) for the first time. The patients were randomly assigned to one of three groups: a blank control group (CG), a placebo group (PG), and a TCM group (TG), with 105 patients in each group, using a random number table method.

Inclusion criteria: (1) Patients who meet the diagnostic criteria for T2DM according to the Chinese Diabetes Prevention and Treatment Guidelines; (2) Patients who are using GLP-1RA for the first time; (3) Patients aged 18 to 60 years; (4) Patients able to clearly express gastrointestinal adverse reactions such as nausea and vomiting; (5) Patients who have not used medications that affect gastrointestinal function; (6) Patients who signed the informed consent form.

Exclusion criteria: (1) Patients allergic to GLP-1RA drugs; (2) Patients with diabetic emergencies (such as hyperosmolar coma, diabetic ketoacidosis, or lactic acidosis); (3) Patients with cancer, pregnancy, lactation, or psychiatric disorders; (4) Patients with primary or secondary digestive system diseases; (5) Patients with severe liver, kidney, heart, metabolic diseases, autoimmune diseases, or hematologic disorders; (6) Patients with local skin damage, allergies, scarring, severe edema, or abnormal local sensations.

Withdrawal and termination criteria: (1) Patients who request to withdraw from the study for any reason; (2) Patients who violate the clinical observation protocol, with missing or incomplete data that affect efficacy or safety assessments; (3) Patients who experience severe adverse events related to the study; (4) Patients who use medications that modify gastrointestinal function during the study [7] [8] [9] [10].

### Sample size calculation

This study employed a wholly randomised controlled design, with the primary outcome measure being the incidence of gastrointestinal ARs associated with GLP-1 receptor agonists. The control group (CG) was not included in the initial comparison. It was anticipated that the incidence of gastrointestinal ARs in PG would be 22%, while the incidence in the TCM nursing group would be 6%. The sample size ratio between the two groups was 1:1, with a significance level ( ) of 0.05 and a power (1-β) of 0.1. The sample size calculation is as follows:


(1) n=\frac{2\overline{pq}(Z_\alpha +Z_\beta )^{2}}{(p1-p2)^{2}}

In this context, n represents the sample size for each group, while Z and Zβ need to be obtained from statistical tables. Typically, is set at 0.05, with a two-tailed Z value, resulting in Z0.05=1.96. For β, when considering a one-tailed test with a power (test efficacy) of 0.9, Zβ equals 1.28. In this study, Z and Zβ were thus 1.96 and 1.28, respectively. Here, p and p represent the incidence rates of gastrointestinal adverse reactions in the TCM nursing and placebo groups, with p =0.06 and p =0.22. The values of p and p denote their respective means, while 1 - p and 1 - p represent the means of the complementary probabilities, calculated as =0.14 and =0.86. These values are then incorporated into the following equation:


(2) \frac{2\times 0.14\times0.86\times(1.96+1.28)^{2}}{(0.22-0.06)^{2}}\approx 99

Considering a potential dropout rate, the sample size was further increased by 5%. Therefore, the target sample size for each group was 105 patients, resulting in a total of 315 participants.

### Intervention methodologies

Patients in CG received standardised treatment supplemented with psychological care and health education according to the conventional nursing plan.

Patients in PG were given a placebo that matched the appearance of the TCM acupoint application, with flour as the main ingredient. This group received the standard treatment and the placebo, following the same procedural steps as the TCM nursing group.

The TCM nursing group received routine treatment and care and acupoint application therapy. The specific method employed was the herbal application technique, using the prescription based on Liangfu Wan as the primary formula, which includes key ingredients such as Zijingpi (Acanthopanax gracilistylus), Duhuo (Angelica pubescens), Chishaoyao (Paeonia lactiflora), Baizhi (Angelica dahurica), and Shichangpu (Acorus tatarinowii). The acupoints selected were Hegu, Zhongwan, Shenque, and Neiguan. The operational steps included assisting the patient in a comfortable position, exposing the application site, cleansing the local skin, and applying the herbal plaster to the abovementioned acupoints. The plaster was to be used for 4 to 6 hours and removed thereafter, with daily applications for 7 days constituting one treatment course. The operational standards for the herbal acupoint application nursing technique are detailed in Table 1.

**Table 1:** Protocol for TCM acupoint application nursing procedures.

Item	Content
Purpose of operation	Prepare the medication in a paste form for application on specific body areas or acupoints, achieving objectives such as promoting meridian circulation and regulating organ functions.
Procedure steps	Verify the medical order.
	Assess the patient:1. Current primary symptoms, clinical manifestations, medical history, and history of drug allergies.2. Patient’s constitution and local skin condition.
	Prepare instruments and wash hands.
	Prepare the environment by cleaning the treatment table, cart, and tray.
	Prepare the necessary materials: treatment tray, medication prepared according to the doctor’s orders, saline cotton balls, and gauze.
	Gather all materials and bring them to the bedside, verify the patient, and provide explanations.
	Assist the patient in a comfortable position, expose the application area, and ensure warmth and pri- vacy.
	Select and locate acupoints according to medical orders and the patient’s condition.
	Remove any existing patches, clean the skin, eliminate traces of previous applications, and apply the new patch to the acupoint for 4–6 hours.
	Upon completion, assist the patient in dressing, arrange a comfortable position, tidy the bed unit, and communicate any precautions.
	Organise materials, wash hands and document the procedure.
	Assess proficiency in the operation.
Precautions	1. Closely observe the local skin condition; if redness, rashes, blisters, itching, or erosion occurs, dis- continue use and promptly report to the physician for further management.<br>2. During the application, monitor for any loosening, displacement, or detachment of the patch and evaluate its effectiveness.

During the trial, any ARs that occur should be promptly recorded and reported. Based on causal assessment of relevant indicators, the relationship between ARs and the medications involved in the study protocol should be accurately determined, with appropriate measures taken. (1) Skin damage: immediate cessation of treatment was required. The area, extent, and severity of the skin damage should be assessed, and the physician should be notified. Following medical advice, the area should be cleaned with saline, and the skin should be kept clean and dry. For significant pain, a hydrocolloid dressing (transparent film) may be applied to protect the area, preventing bacterial invasion and promoting wound healing. If the dressing gets milky translucent colour, it needs to be changed, and the healing of the skin should be monitored. (2) Skin allergies: treatment should be stopped immediately. The area and severity of the allergic reaction should be assessed, and the physician should be informed. Following medical advice, antihistamine treatment should be administered, and patients should wear cotton clothing while avoiding scratching the affected area. Improvements should be observed in the allergic condition. (3) Blisters: treatment should be stopped immediately. The size of the blisters should be assessed, and the physician should be notified. Following medical advice, blisters smaller than 5 mm should be disinfected with PVP iodine, and the area should be kept clean and dry. For blisters larger than 5 mm, sterile syringes may be used after disinfection to aspirate the fluid from the blister while maintaining local cleanliness and dryness. Hydrocolloid dressings can also be applied to protect the area, and the healing process should be monitored [11] [12] [13].

### Observational indicators

For demographic data, a demographic and socioeconomic data questionnaire was used to collect relevant information on gender, age, weight, waist circumference, disease duration, household monthly income, and education level for all three patient groups.

Efficacy assessment criteria were applied. A disease-related data questionnaire was employed to gather pre- and post-treatment data on routine blood parameters (haemoglobin (Hb), white blood cell (WBC) count, erythrocyte sedimentation rate (ESR)), and renal function indicators (blood urea nitrogen (BUN), serum creatinine (Cr)).

Gastrointestinal symptom evaluation was implemented. The TCM Gastrointestinal Symptom Scoring Scale was used to periodically assess patients’ fasting BG (FBG), postprandial BG at 2 hours (2hPBG), glycated Hb (HbA1c), fasting INS (FINS), INS β secretion (HOMA-β), and insulin resistance (IR) (HOMA-IR) to determine whether these factors influence BG control.

A safety assessment was made. The TCM Syndrome Scoring Scale was utilised to record any AR events during treatment in all three patient groups, including symptoms such as nausea, vomiting, abdominal distension, reduced appetite, hypoglycemia, and reflux.

### Statistical methodologies

Statistical analysis of the study data was performed employing SPSS version 22.0. Normally distributed continuous variables were indicated as mean ± standard deviation (x̄ ± s), while categorical data were presented as frequencies and percentages (%). The Mann-Whitney U test was utilised for non-normally distributed continuous variables for inter-group comparisons. A one-way analysis of variance was employed for normally distributed continuous variables. Chi-square tests were used to compare categorical data. A two-tailed significance level of P<0.05 was considered statistically significant.

## Results

### General data

**Figure 1:**
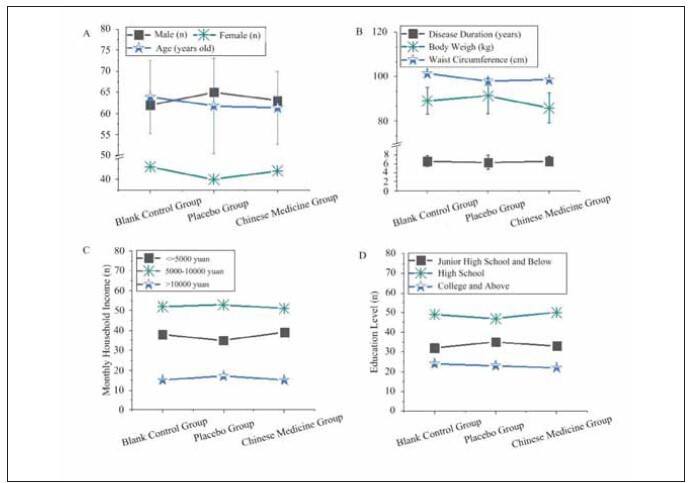
Comparison of general characteristics among the three patient groups.<br>(A: gender and age; B: weight, waist circumference, and disease duration; C: household monthly income; D: education level)

The general characteristics of the patients are presented in Figure 1. The gender, age, weight, waist circumference, disease duration, household monthly income ( 5,000 RMB, 5,000-10,000 RMB,>10,000 RMB), and education level (middle school and below, high school, college and above) differed slightly among CG, PG, and TG when compared pairwise (P>0.05).

### BG levels

**Figure 2:**
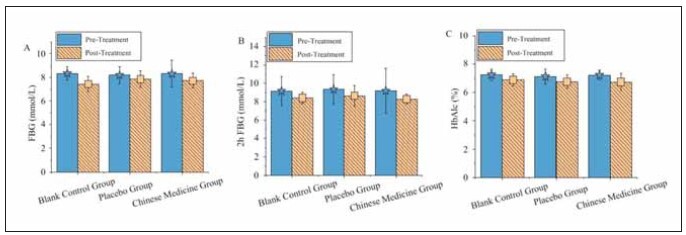
Comparison of BG levels before and after treatment among the three patient groups.<br>(A–C represent FBG, 2hPBG, and glycated Hb, respectively)

The BG levels are illustrated in Figure 2. Compared to baseline, the FBG, postprandial BG at two hours, and glycated Hb levels showed slight reductions in all three groups after treatment; however, these differences were not statistically significant (P>0.05). Furthermore, the FBG, postprandial BG at two hours and glycated Hb levels in the TG did not differ significantly from those in the CG and the PG (P>0.05).

### INS indicators

**Figure 3:**
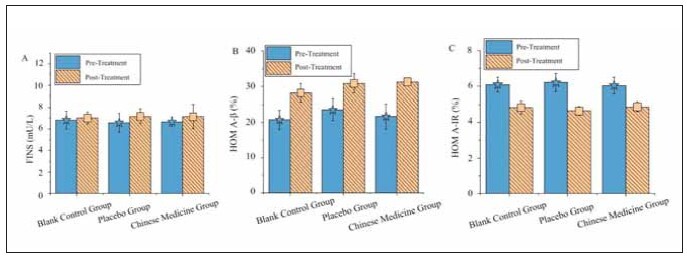
Comparison of INS parameters before and after treatment among the three patient groups.<br>(A–C represent FINS, INS β secretion, and IR, respectively)

In Figure 3, the TG, the post-treatment levels of FINS, β-cell insulin secretion, and IR did not show statistically significant differences compared to the CG and the PG (P>0.05).

### Blood routine indicators

**Figure 4:**
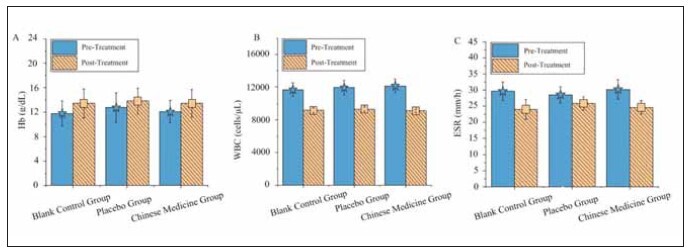
Comparison of blood routine parameters before and after treatment among the three patient groups.<br>(A–C represent Hb, WBC count, and ESR, respectively)

In Figure 4, in the TG, the post-treatment levels of Hb, WBC count, and ESR did not show statistically significant differences compared to the CG and the PG (P>0.05).

### Renal function indicators

**Figure 5:**
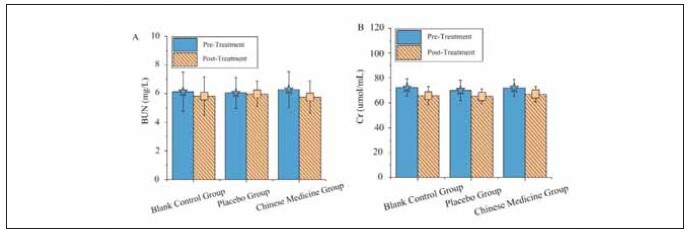
Comparison of renal function indicators before and after treatment in three groups.<br>(A and B represent BUN and serum Cr, respectively)

The renal function indicators are illustrated in Figure 5. In the TG, BUN and serum Cr post-treatment levels did not show statistically significant differences compared to the CG and the PG (P>0.05).

### ARs

**Figure 6:**
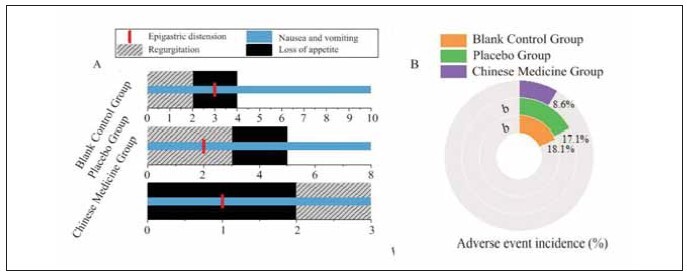
Comparison of adverse reaction events among the three groups.<br>(A includes the number of cases of nausea and vomiting, gastric fullness, appetite reduction, and regurgitation; B represents the incidence of adverse reactions). Note: b indicates a statistically significant difference compared to the TG (P<0.05).

In Figure 6, the TG reported 3 cases of nausea and vomiting, 1 case of gastric fullness, 2 cases of appetite reduction, and 3 cases of regurgitation. The PG had 8 cases of nausea and vomiting, 2 cases of gastric fullness, 5 cases of appetite reduction, and 3 cases of regurgitation. The CG experienced 10 cases of nausea and vomiting, 3 cases of gastric fullness, 4 cases of appetite reduction, and 2 cases of regurgitation. The incidence of adverse reactions in the TG (8.57%) was significantly lower than that in the PG (17.14%) and the CG (18.1%), with a statistically significant difference (P<0.05).

## Discussion

T2DM is a chronic disease with far-reaching effects on patients. Prolonged hyperglycemia can lead to various complications, potentially subjecting patients to psychological stress, such as anxiety and depression. Therefore, comprehensive intervention and regular follow-up are crucial for reducing the risk of complications and improving overall patient health [14] [15]. When patients with T2DM initially use GLP-1RAs, they may face multiple hazards and complications, including the risk of hypoglycemia, gastrointestinal discomfort (such as nausea, vomiting, and diarrhoea), increased risk of pancreatitis, weight changes, injection site reactions, and potential impacts on renal function [16]. Consequently, it is essential to prevent ARs during treatment. This study selected 315 patients from the Department of Endocrinology and Metabolism at our hospital who were hospitalised between July 2021 and October 2023 and received GLP-1RAs for the first time. The patients were randomly assigned to three groups using a random number table: a blank CG (standardised treatment + routine nursing), a PG (placebo + routine nursing), and a TG (TCM Liangfu pill acupuncture point application + routine nursing), with 105 patients in each group. In the TG, the post-treatment levels of FBG, postprandial BG at two hours, and glycated Hb did not show statistically significant differences compared to the CG and the PG (P>0.05). This finding contrasts with the arguments presented by Werida et al. [17] regarding the combined administration of omega-3 fatty acids and glimepiride in controlling BG levels in patients with type 1 diabetes mellitus. It indicates that applying Liangfu Pills via acupoint plaster, in conjunction with standard care, does not significantly affect BG control in patients with T2DM.

FINS, β-cell insulin secretion, and IR are essential indicators for assessing pancreatic function and glucose metabolism [18]. The analysis in this study revealed that the post-treatment levels of FINS, β-cell insulin secretion, and IR in the TG did not show statistically significant differences compared to the CG and the PG (P>0.05), further indicating that the application of Liangfu Pills via acupoint plaster did not significantly impact pancreatic function and glucose metabolism in these patients. Regarding routine blood parameters, the post-treatment levels of Hb, WBC count, and ESR in the TG also showed no statistically significant differences when compared to the CG and the PG (P>0.05), suggesting that the effects of the acupoint plaster did not extend to inflammatory responses or nutritional status. Comparative analysis of renal function indicators showed that the post-treatment levels of BUN and serum Cr in the TG did not differ significantly from those in the CG and the PG (P>0.05). This suggests that applying Liangfu Pills via acupoint plaster does not considerably impact renal function in diabetic patients [19]. In terms of safety, the incidence of adverse reactions in the TG (8.57%) was substantially lower than that in the PG (17.14%) and the CG (18.1%), with a statistically significant difference (P<0.05). This indicates that adding Liangfu Pills via acupoint plaster to GLP-1 receptor agonist therapy can effectively reduce the risk of adverse reactions. It suggests that the Liangfu Pill may improve patient tolerance through its mild effects and regulatory mechanisms, thereby decreasing the adverse reactions of GLP-1 receptor agonist therapy [20]. These findings strongly support the use of TCM as an adjunctive treatment strategy in clinical practice, enhancing the safety and efficacy of the treatment.

## Conclusion

This study evaluated the impact of traditional Chinese medicine (TCM) Liangfu Pills administered via acupoint plaster in patients with type 2 diabetes mellitus (T2DM) initiating GLP-1 receptor agonist therapy. The results indicate that while the intervention did not significantly improve glycemic control, pancreatic function, or haematological parameters, it notably reduced the incidence of gastrointestinal adverse reactions. These findings suggest that TCM acupoint therapy could be a valuable adjunct to standard treatment, enhancing patient tolerance and safety. However, given the study’s limitations – including sample size and follow-up duration – future research should explore its long-term effects and potential mechanisms to optimise diabetes management strategies.

## Dodatak

### Author contributions

All authors contributed to the study’s conception and design. Songsong Zheng and Diyi Zhou performed material preparation, data collection, and analysis. Fangfang Chen and Jiandi Zheng wrote the first draft of the manuscript, and all authors commented on previous versions. All authors read and approved the final manuscript.

### Ethical Approval Statement

This study has been approved by the Ethics Committee of Hangzhou Red Cross Hospital (approval number: [2021] Research Review No. (252)), and all experimental procedures comply with the Declaration of Helsinki and relevant ethical regulations. Clinical data and biological samples from patients are used solely for research purposes, and the collection of all samples follows the requirements of the ethics committee.

### Informed consent

The authors affirm that human research participants provided informed consent to publish the images in Figures 1A, 1B, and 1C.

The participant consented to submitting the case report to the journal.

Patients signed informed consent regarding publishing their data and photographs.

### Availability of data and materials

The datasets generated during and/or analysed during the current study are available from the corresponding author upon reasonable request.

### Funding

The research is supported by Zhejiang Provincial Science and Technology Program of Traditional Chinese Medicine (No. 2023ZLl555); Hangzhou Red Cross Hospital in-Hospital Youth Fund Project (No. HHQN2021008).

### Conflict of interest statement

All the authors declare that they have no conflict of interest in this work.
